# Pannexin- and Connexin-Mediated Intercellular Communication in Platelet Function

**DOI:** 10.3390/ijms18040850

**Published:** 2017-04-17

**Authors:** Filippo Molica, Florian B. Stierlin, Pierre Fontana, Brenda R. Kwak

**Affiliations:** 1 Department of Pathology and Immunology, University of Geneva, 1211 Geneva, Switzerland; florian.stierlin@gmail.com (F.B.S.); brenda.kwakchanson@unige.ch (B.R.K.); 2 Department of Medical Specializations, Cardiology, University of Geneva, 1211 Geneva, Switzerland; 3 Geneva Platelet Group, University of Geneva, 1211 Geneva, Switzerland; pierre.fontana@hcuge.ch; 4 Division of Angiology and Haemostasis, Geneva University Hospitals, 1211 Geneva, Switzerland

**Keywords:** platelet aggregation, collagen, connexins, pannexins

## Abstract

The three major blood cell types, i.e., platelets, erythrocytes and leukocytes, are all produced in the bone marrow. While red blood cells are the most numerous and white cells are the largest, platelets are small fragments and account for a minor part of blood volume. However, platelets display a crucial function by preventing bleeding. Upon vessel wall injury, platelets adhere to exposed extracellular matrix, become activated, and form a platelet plug preventing hemorrhagic events. However, when platelet activation is exacerbated, as in rupture of an atherosclerotic plaque, the same mechanism may lead to acute thrombosis causing major ischemic events such as myocardial infarction or stroke. In the past few years, major progress has been made in understanding of platelet function modulation. In this respect, membrane channels formed by connexins and/or pannexins are of particular interest. While it is still not completely understood whether connexins function as hemichannels or gap junction channels to inhibit platelet aggregation, there is clear-cut evidence for a specific implication of pannexin1 channels in collagen-induced aggregation. The focus of this review is to summarize current knowledge of the role of connexins and pannexins in platelet aggregation and to discuss possible pharmacological approaches along with their limitations and future perspectives for new potential therapies.

## 1. Introduction

Atherosclerosis is an inflammatory disease affecting medium-to-large sized arteries and leading to the formation of atheroma. Plaque formation starts during the early years [1] favored by risk factors inducing endothelial dysfunction as an initiating stage of the disease. Atherosclerosis evolves through different stages, sometimes leading to the formation of unstable atherosclerotic plaques prone to rupture and to acute ischemic events [2] via the formation of a platelet-rich thrombus. Strong links between platelet aggregation and atherogenesis have been shown and will be discussed here.

The initial vascular lesion increases endothelium permeability, adhesiveness for leukocytes and platelets, and it induces procoagulant properties [3]. Inflammation stimulates smooth muscle cell accumulation at the vascular lesion and release of mediators (cytokines, growth factors) from monocyte-derived macrophages and T lymphocytes. This further enhances the development of atherosclerotic plaques through a vicious circle (recruitment of more T lymphocytes, monocytes and smooth muscle cells) [4,5]. In parallel, lipids accumulate in the lesion, are taken up by macrophages, lipid-laden macrophages die and a lipid core is formed. A fibrous cap, composed principally of smooth muscle cells that originate from the media, also develops with variable levels of stability depending on the quantity of extracellular matrix components (mainly collagen) produced by these cells [6].

Rupture of the fibrous cap and erosion of the intimal surface of the atheroma are two mechanisms leading to exposure of the plaque’s thrombogenic material to the blood [2]. These mechanisms are mediated by different protagonists such as cytokines, matrix metalloproteinases, or endothelial Toll-like receptors [7,8]. Thrombus formation is a rapid process in which platelets play a major role by going through different steps: platelet adhesion, activation and aggregation. Various ion channels such as P2X_1_ purinergic receptors, Orai1 store-operated channels, connexin and pannexin channels as well as α-amino-3-hydroxyl-5-methyl-4-isoxazole-propionate (AMPA) and *N*-methyl-d-aspartate (NMDA) glutamate receptors receive increased attention as potential regulators of platelet function and thrombus formation [9,10]. In this review, we describe the major steps leading to thrombus formation, which will be explained separately for didactic reasons, with a particular focus on the role of connexins and pannexins in platelet aggregation.

## 2. Platelet Function: From Plaque Rupture to Thrombus Formation

### 2.1. Platelet Adhesion and Activation

With rupture of the fibrous cap, the highly thrombogenic sub-endothelial matrix is exposed to circulating platelets [2]. This contact initiates primary hemostasis, with the first step being platelet adhesion to the site of vascular injury.

**Figure 1:**
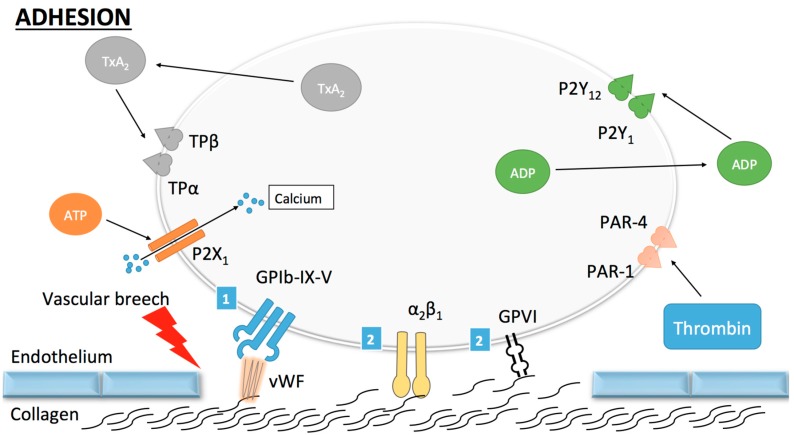
Platelet adhesion. (1) Indirect adhesion pathway: once circulating platelets are exposed to the thrombogenic subendothelial matrix, von Willebrand factor (vWF) binds to collagen and undergoes a conformational change permitting its subsequent binding to the GPIb-IX-V (GP, glycoprotein) complex. This mechanism allows for the tethering of platelets to the vascular wall and subsequent stronger adhesion through the direct pathway; (2) Direct adhesion pathway: once platelets are slowed down, binding of collagen to GPVI and α_2_β_1_ occurs and promotes stable platelet adhesion to the vascular wall. Other receptors (ADP, ATP and thromboxane A_2_) are shown here but do not play a role in platelet adhesion.

Two pathways are known to participate in platelet adhesion, namely, the direct and the indirect pathways [11]. In conditions of rapid blood flow (such as arterial flow), a strong and rapid connection between the vascular wall and platelets needs to be established. This is the role of the indirect pathway, which works principally through one glycoprotein (GP) called von Willebrand factor (vWF). The adjective “indirect” refers to the necessity of vWF to establish binding between collagen and platelets. vWF is found in the sub-endothelial matrix, in circulating blood as an inactive form, in the Weibel Palade bodies of endothelial cells and in α-granules of platelets [12]. When collagen is exposed, vWF binds to it and undergoes conformational changes [13]. These changes occur at high shear stress and uncover the binding site for the GPIb-IX-V complex on vWF [11]. The binding of vWF to GPIb-IX-V allows the tethering of platelets onto the vascular wall at the site of vascular injury allowing platelet adhesion to collagen (Figure 1). Of note, in order to bind to collagen, vWF must first be in an immobilized form, which implicates a process not yet fully understood [14].

The direct pathway corresponds to the interaction between sub-endothelial structures such as collagen with specific receptors on the surface of platelets. Collagen receptors are named α_2_β_1_ and GPVI [11]. The direct pathway plays its role in conditions of low blood flow and reinforces platelet–platelet adhesion. Through this mechanism, a monolayer of activated platelets is created, which permits the further recruitment and the growth of the platelet plug [15].

Once the adhesion complex is stable, platelet activation takes place involving a shape change as well as an initial secretion of granules. The transformation of the integrin α_IIb_β_3_ receptor from its resting low-affinity state to a high affinity state ends the activation process, subsequently mediating platelet aggregation [16]. Human platelets can be activated by numerous agonists like thrombin, which is produced in small quantities after the binding of tissue factor to coagulation factors at the surface of activated platelets [17]. This allows for further activation and recruitment of resting platelets through the cleavage of protease-activated receptor (PAR)-1 and PAR-4 [18], as well as for the subsequent platelet shape change and intra-cytoplasmic calcium release [15,19]. However, despite the important role of thrombin, collagen remains the central actor in the context of atherothrombosis.

#### 2.1.1. Collagen

**Figure 2:**
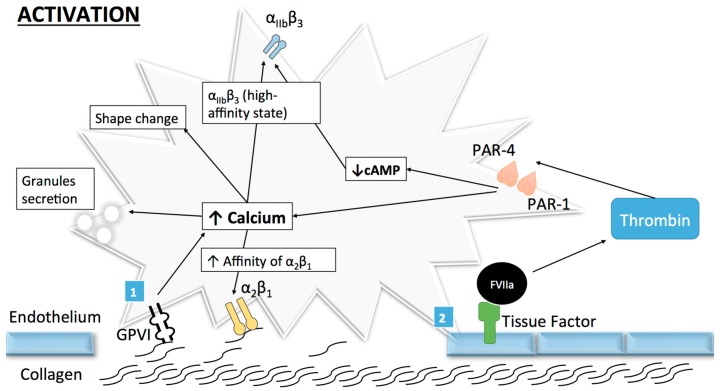
Platelet activation: Once platelets arrest on the vascular wall, activation occurs under the control of several molecules, among which collagen and thrombin. (1) Collagen-induced platelet activation: binding of sub-endothelial collagen fibrils to platelet receptor GPVI induces cross-linking and subsequent activation of GPVI/FcR-γ-chain complex (an intracellular part of GPVI). This activation leads, through a cytoplasmic phosphorylation cascade, to an increase in cytoplasmic calcium concentration and a subsequent platelet shape change, granule secretion and/or increase in affinity of platelet surface receptors. The increase in affinity of α_2_β_1_ enhances its binding to collagen. The final step in platelet activation is the transformation of α_IIb_β_3_ from its low to its high-affinity state in order to promote platelet aggregation; (2) Thrombin mediated platelet activation: tissue factor in its active form binds to and activates FVII, which promotes an initial generation of small quantities of thrombin and a later burst in thrombin generation (through a cascade comprising other coagulation factors). Thrombin then favors platelet activation by cleavage of protease-activated receptors (PARs) also leading to increased calcium concentration and decreased cyclic adenosine monophosphate (cAMP).

Because of its major role in platelet activation and adhesion, platelet–collagen interaction is a major focus of research. Platelets can bind collagen through several receptors such as collagen GPVI and the integrin receptors α_2_β_1_ and α_IIb_β_3_ (Figure 2). While GPVI participates in platelet activation, α_2_β_1_ has a more important role in platelet adhesion and α_IIb_β_3_ in aggregation. Therefore, GPVI and α_2_β_1_ will be discussed in the following section.

##### Glycoprotein VI

The GPVI receptor is a 319 amino-acid GP expressed on platelets and megakaryocytes [20] but also on other cells [21]. GPVI responds exclusively to collagen as proven in patients with auto-immune thrombocytopenia leading to a deficiency in this receptor [22]. Structurally, GPVI is a complex composed of an extracellular part with two immunoglobin-like domains (GPVI molecules) non-covalently bound to an intracellular part, the Fc receptor (FcR) γ-chain [23,24]. When bound to fibrinous collagen, cross-linking and subsequent activation of GPVI/FcR γ-chain complex occurs and leads to Src kinase-dependent (Fyn and Lyn) phosphorylation of an Immunoreceptor Tyrosine-based Activation Motif (ITAM) present in the FcR γ-chain portion [25]. This favors the recruitment and the auto-phosphorylation of Spleen tyrosine kinases (Syk) and the subsequent phosphorylation of several intracellular effectors such as phospholipase Cγ2 (PLCγ2) or phosphoinositide-3 kinase (PI3-kinase) [26].

PLCγ2 catalyzes the formation of the second two messengers, namely, 1,4,5-trisphosphate (IP_3_) and 1,2-diacylglycerol (DAG) [27], from phosphatidylinositol 4,5-bisphosphate [28,29]. IP_3_ induces a rapid raise of cytoplasmic calcium concentration through mobilization of intracellular stores (from the platelet dense tubular system) and influx from extracellular space leading to platelet activation [30]. DAG activates protein kinase C (PKC) [31], which leads to thromboxane A_2_ (TxA_2_) release [32].

GPVI activates the PI3-kinase, resulting in phosphatidylinositol 3,4,5-trisphosphate (PIP3) production [26], which is thought to play a role in PLCγ2 regulation [33]. The specific affinity of GPVI for collagen, and its compulsory action during collagen-induced platelet aggregation, makes GPVI a major signaling receptor for collagen in platelets.

##### Integrin α_2_β_1_

α_2_β_1_ (also known as GPIa-IIa) is an integrin expressed on several cell types including lymphocytes [34] and fibroblasts [35], and is required for platelet adhesion to the vascular wall [36]. It works as an extracellular receptor for collagen and/or laminins depending on the cell type expressing it [37]. Similar to all other integrins, α_2_β_1_ displays a heterodimeric structure composed of an α- (α_2_) and a β-subunit (β_1_) with a transmembrane domain and intracellular domains [38].

α_2_β_1_ exists in a low-affinity state with its extracellular domain into a close conformation. It is activated by GPVI to form a high-affinity state (with conformational changes in extracellular domains) thus permitting stronger binding to collagen [11]. α_2_β_1_ activation is induced by an “inside-out” process, where proteins bind to the intracellular α- and β-tails of the receptor and provoke a conformational change in the extracellular domain leading to the high-affinity state [39,40]. As previously mentioned, numerous proteins can interact with the cytoplasmic tails of integrins and talins, which are strong activators for α_2_β_1_ [39]. Talin, a cytosolic protein abundantly found in platelets [41], is composed of a head domain (talin-H) and a rod domain (talin-R). The head domain can bind to the integrin β cytoplasmic domain [42] and induce a disruption in salt bridges from the cytoplasmic tails, thus exposing α_2_β_1_ extracellular binding site, such as the α_2_-I domain [40] and enhancing α_2_β_1_ affinity for collagen [42]. Of note, other agonists such as ADP, thrombin or collagen-related peptide (CRP) have been shown to activate α_2_β_1_ from its low to its high-affinity state [43]. Given the various kinetics of α_2_β_1_, Jung and Moroi [43] proposed a multistate model of α_2_β_1_ activation where three affinity states exist. However, whether the process occurs sequentially or not is yet unknown.

α_2_β_1_ is also thought to generate intracellular signals via an “outside-in” process [44]. This integrin is known for having a supplementary inserted domain (I-domain) on the α_2_ globular head containing a high-affinity binding site for collagen (GFEOGR motif) [45] and a metal ion-dependent adhesion site (MIDAS) [46]. MIDAS permits a magnesium-dependent binding to collagen [34], which induces conformational changes in the aforementioned I-domain [38]. It is unclear whether collagen binding to α_2_β_1_ results in the activation of intracellular signaling pathways, but several observations support such a hypothesis. Jararhagin, a specific inhibitor of α_2_β_1_ acting by cleavage of the GPIa subunit [47], has been shown to inhibit α_2_β_1_ activation and tyrosine activation in response to collagen [48]. However, this effect may be surmounted by increasing collagen concentrations [49]. Moreover, a selective peptide for α_2_β_1_ [50] has been developed, which did not succeed in stimulating tyrosine phosphorylation [51], an expected intracellular effect. It is likely that GPVI and α_2_β_1_ operate synergistically in both platelet adhesion and activation and that GPVI is not able to control all aspects of collagen-induced platelet activation and aggregation. This observation is supported, among others, by the fact that collagen-binding to α_2_β_1_ is already possible in the low-affinity (non-activated) state [52].

### 2.2. Platelet Aggregation, Thrombus Growth and Stabilization

**Figure 3:**
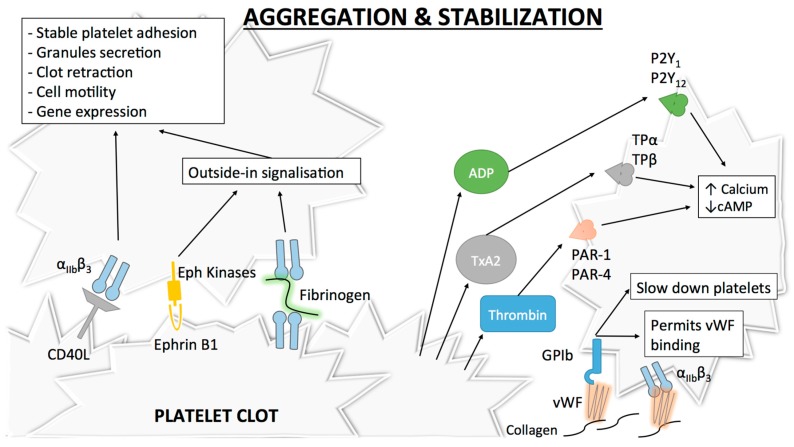
Platelet aggregation and clot stabilization. Platelet aggregation: α_IIb_β_3_ high-affinity state permits its binding to fibrinogen thus initiating the “outside-in” signaling process and promoting stable aggregation. Extension and stabilization of the platelet clot: Through paracrine secretion of platelet agonists (notably adenosine diphosphate (ADP), thromboxane A_2_ (TxA_2_) and thrombin), extension of the platelet plug occurs. These agonists act on different receptors with various signaling mechanisms but with the same final effect, i.e., increased intracellular calcium and decreased cAMP concentrations. This promotes platelet activation, aggregation, recruitment of more platelets and shape change. Inter-platelet close contact allows stronger platelet binding and thus stabilisation of the clot. Several molecules and receptors are known to participate in this process such as CD40-ligand (CD40L), ephrin B1 ligand and ephrine kinases receptor, or α_IIb_β_3_.

The final step of platelet activation is to promote integrin α_IIb_β_3_ (previously known as GPIIb-IIIa) change from its low- to its high-affinity state, which allows irreversible α_IIb_β_3_ binding to fibrinogen and vWF and consequently leads to stable platelet aggregates [53,54,55] (Figure 3). α_IIb_β_3_, the most abundant integrin on platelet surface [56], is a heterodimer composed of an extracellular part containing two subunits (α_IIb_ and β_3_) linked by non-covalent bonds, a single transmembrane domain and a small cytoplasmic domain [54,57,58]. Similar to α_2_β_1_, this conformational change is mediated through an “inside-out” mechanism, but α_IIb_β_3_ is also able to function via an “outside-in” process [59]. As α_IIb_β_3_ is a central player in platelet aggregation, numerous proteins influence its conformational change and more than 20 have already been described [60]. Of these proteins, talin is thought to be of particular importance for α_IIb_β_3_ activation [42]. Some other proteins named Kindlins (and particularly Kindlin-3) have also been described as regulators of talin–integrin interaction and act as stimulators for α_IIb_β_3_ activation [61]. Given the numerous molecules involved, the complete pathway to α_IIb_β_3_ activation and modulation is still not clearly understood. The actual consensus is that intracellular calcium and DAG levels stimulate the production of Rap1, a small GTPase acting as a potent regulator of integrin activation [62]. Rap1 needs an effector molecule called the Rap1-GTP-interacting adaptor molecule (RIAM) to modulate the interaction between talin and Rap1-GTP [63] and activate the “inside-out” signaling. Of note, as Rap1 depletion does not lead to complete abolishment of platelet aggregation, other activation pathways independent of this protein may be involved [64].

Similar to other integrins, “outside-in” signaling also exists with α_IIb_β_3_. After α_IIb_β_3_ activation, conformational changes in its transmembrane and cytoplasmic domains occur [65]. Microclustering also occurs, which is an imprecisely defined process where receptors cluster at the cell surface [66]. These modifications induce several intracellular events leading to stable platelet adhesion, granule secretion, clot retraction and also cell motility or gene expression [67,68]. A well-known event in this signaling cascade is the binding of a G-protein subunit (Gα13) to the α_IIb_β_3_ cytoplasmic domain of β3, which stimulates the activation of Src family kinases (SFKs) [69]. SFKs are implicated in cell migration and spreading [70] and participate in the outside-in signaling through three different mechanisms. The first mechanism involves the phosphorylation of two tyrosine-site motives in the β3 cytoplasmic tail [71], which negatively regulates talin binding [72] (thus playing a role in clot dissemination), and protection from calpain cleavage (which normally inhibits cell spreading) [73]. A second mechanism involves the stimulation of a GTPase activating protein [74] by a c-Src tyrosine kinase in the β3 cytoplasmic tail of α_IIb_β_3_, which permits inhibition of RhoA [75], a GTPase regulating cell contractility and the formation of protrusive structures for cell spreading [76]. Finally, SFKs can also activate Syk [77], which phosphorylates FcγRII, thus leading to PLCγ2 activation (and therefore platelet activation) through the assembly of an intracellular complex [78].

After platelet activation and once α_IIb_β_3_ is in its high affinity state, platelet aggregation as well as thrombus extension and stabilization occur. Extension is possible via paracrine action of platelet soluble agonists, mainly thrombin (as described above) but also ADP and TxA_2_, which induce cAMP decrease and cytoplasmic calcium increase via the activation of their respective receptors P2Y_1_/P2Y_12_ (for ADP) and TPα/TPβ (for TxA_2_) [15,79,80,81,82,83,84].

The freshly formed platelet plug needs stabilization and strong platelet contacts to avoid its dissolution. Different signaling mechanisms strengthen thrombus stability. Of these mechanisms, the “outside-in” process linked to α_IIb_β_3_ [67] or the binding between α_IIb_β_3_ and CD40L, a member of the tumor necrosis factor family of ligands that is expressed on activated platelets, play a major role [85]. Other inter-platelet mechanisms promoting clot stabilization include the binding of Eph receptor kinase to its ephrin B1 ligand (which also participate in “outside-in” process) [86] (Figure 3).

### 2.3. Towards New Antiplatelet Agents

The currently used antiplatelet agents target several key actors of platelet function such as TxA_2_ generation (aspirin) or the P2Y_12_ receptor (anti-P2Y_12_ drugs). The main adverse event related to these drugs is bleeding and huge efforts are being done to identify new drug targets, allowing a fine-tuning of platelet function with a more favorable benefit/risk ratio.

Among potential targets [87], GPVI as well as some types of membrane channels are of particular interest [88]. Indeed, various calcium channels such as the ATP-gated P2X_1_ purinergic receptors, the Orai1 store-operated channels, Kv1.3 voltage-gated channels as well as AMPA and NMDA glutamate receptors have been shown to participate in the regulation of platelet function [10]. In the following sections of this review, we will exclusively focus on the role of two other types of membrane channels, pannexins (Panx) and connexins (Cxs), of which the essential role in platelet function is increasingly recognized.

## 3. Connexins and Pannexins

**Figure 4:**
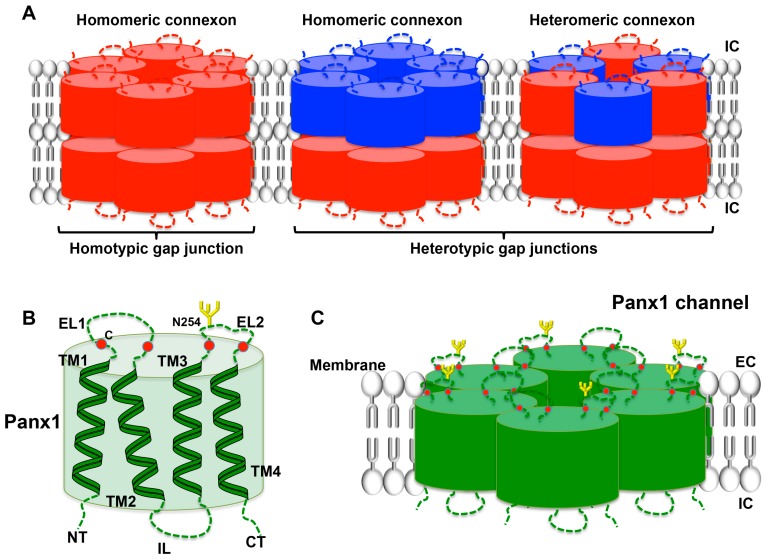
Connexin gap junctions and Pannexin1 channels. (**A**) Homotypic or heterotypic gap junction channels assemble in the extracellular space by the docking of two homomeric or heteromeric connexons from neighboring cells. Connexons are formed by the hexamerization of six Cx subunits (e.g. Cx37 in red and Cx43 in blue) and may function as hemichannels under particular conditions; (**B**) Similar to connexins, pannexins, including Panx1, are membrane proteins composed of four transmembrane helices (TM1-TM4), two extracellular loops (EL1 and EL2), one intracellular loop (IL) and an N- and a C-terminal end located in the cytoplasm (NT and CT); (**C**) Six Panx1 subunits assemble into a pannexon. However, due to glycosylation on the N254 residue located in EL2, Panx1 is unable to form gap junctions and functions exclusively as a single-membrane channel.

Cxs are members of a family of proteins encoded by 20 to 21 different mammalian genes, which are expressed in a large variety of tissues [89,90]. Cx genes are classified according to sequence homology and are divided into five subfamilies [91]; cardiovascular Cxs belong mostly to the α subfamily (*GJA*). Cx proteins are named after their specific molecular weight in kDa (for instance, Cx43). The general structure of a Cx consists of four α-helical transmembrane domains (TM1-TM4) and two extracellular loops (EL1 and EL2) that are highly conserved among the family members. The amino-terminal (NT), carboxy-terminal (CT) parts and the intracellular loop (IL), which are all located in the cytoplasm, differ considerably in both length and composition and are nearly unique to each Cx type. Cxs are synthesized in the endoplasmic reticulum (ER) and oligomerize in the ER/Golgi or trans-Golgi network to form hexameric connexons [92,93]. After that, connexons are transported to the plasma membrane along microtubules. When two cells are closely apposed, connexons from one cell can dock with their counterparts in the neighboring cell and form a gap junction channel enabling the intercellular exchange of ions and small messenger molecules up to ~1 kDa. These channels are called homomeric, heteromeric or heterotypic depending on whether they are composed of identical or different Cx isoforms [94] (Figure 4A). Under specific conditions, connexons may function as a hemichannel allowing the permeation of ions and small metabolites such as calcium, ATP, ADP and glutamate [95,96]. A variety of factors regulate Cx channel gating, including voltage, pH, and Ca^2+^, phosphorylation, *S*-nitrosylation and other post-translational modifications [97,98,99]. Recently, increasing evidence points to an important function for the so-called connexin interactome, a protein interacting network with the Cx being the central mediator [100,101]. Maintenance of hemichannel and/or gap junction functionality and integrity is fundamental to conserve intercellular communication and homeostasis in a wide range of tissues. The essential nature of Cxs in tissue homeostasis is further emphasized by the multitude of pathologies that are linked to mutations and polymorphisms in Cx genes [90,102]. The expression and function of Cxs in platelets has only recently attracted research interest.

Panx represent yet another small family of three transmembrane proteins (Panx1–3) that were discovered only in the year 2000 [103]. The *Panx1* and *Panx3* genes are both located on human chromosome 11, while the *Panx2* is located on chromosome 22 [104]. The Panx proteins exhibit a topology analogous to Cxs (Figure 4B), although they share no sequence homology [105,106,107]. Initial characterization of Panx1 oligomerization showed that this Panx forms hexameric single-membrane channels (Figure 4C) [108]. Panx1 is ubiquitously present in human and mouse tissues, whereas Panx2 seems to be predominant in the brain and the central nervous system, and Panx3 is mostly expressed in bones, cartilage and skin [109,110,111]. The general expression pattern of Panx1 likely explains that the vast majority of basic and clinical studies focus on this Panx. Important glycosylation sites are found in the ELs of Panx1 and Panx3 [107,108]. The occupation of these sites likely prevents docking of pannexons (Figure 4C). It is now generally accepted that Panx function as single-membrane channels. Similar to Cx hemichannels, Panx channels allow for intercellular communication by the release of small molecules such as purines that can signal via targeting surface receptors in a paracrine, or even in an endocrine, fashion. However, it is important to keep in mind that Panx channels can be opened at physiological membrane potential and extracellular calcium by several mechanisms: mechanical stretch, phosphorylation by Src kinases, caspase cleavage of its C-terminus and following purinergic P2 receptor stimulation [107,110,112,113,114]. Connexon opening is generally induced under more pathological conditions such as low extracellular calcium or very large depolarizations [115]. As described above, purinergic receptors are expressed in platelets where they are major players in platelet activation. In fact, ADP-dependent activation of metabotropic P2Y_1_ and P2Y_12_ purinergic receptors occurs after platelet stimulation through thrombin, TxA_2_ or collagen. On the other hand, the ionotropic receptor P2X_1_, in response to ATP stimulation, contributes to the increase in intracellular calcium leading to the initial shape change of platelets followed by aggregation [116]. While the role of Cxs and Panx1 channels in the regulation of vascular tone, the immune response and in neurophysiology is increasingly recognized [99,117,118,119], their role in platelet function has only recently been discovered. The next paragraphs summarize these new advances.

### 3.1. Pannexin1 and Platelet Aggregation

**Figure 5:**
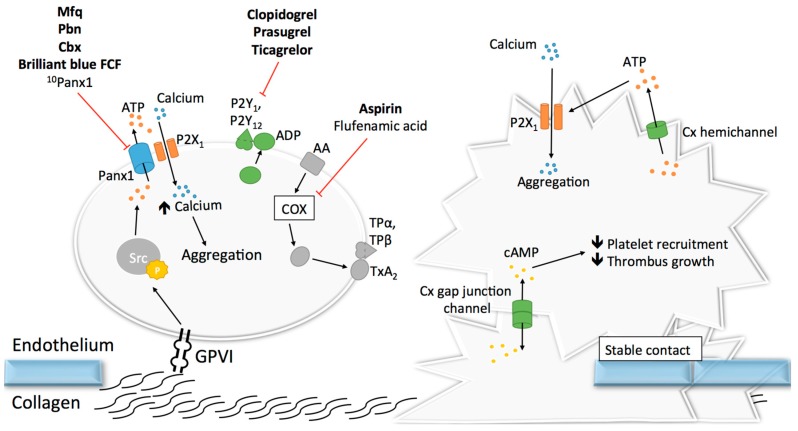
Panx1- and Cx-dependent mechanisms leading to platelet aggregation. Upon GPVI binding to collagen, Src phosphorylation occurs, which permits opening of Panx1 channels and ATP release from platelets. ATP activates P2X_1_ purinergic receptors, enhancing intracellular calcium concentration and therefore platelet activation and aggregation. Apart from classical anti-platelet drugs targeting signaling pathways linked to ADP and thromboxane A2 (TxA_2_) receptors, Panx1 inhibitors (FDA approved compounds are indicated in bold) mefloquine (Mfq), probenecid (Pbn), carbenoxolone (Cbx), Brilliant blue FCF and the specific peptide ^10^Panx1 have demonstrated inhibitory effects on collagen-induced aggregation (see also Table 1). The role of Cxs in platelet aggregation remains to be further clarified. Two pathways are thought to play a role in platelet aggregation. The first one would occur after stable platelet contact is established, allowing formation of gap junctions and cAMP trafficking between adjacent platelets. cAMP has a regulatory role, inhibiting further platelet recruitment and thrombus growth. The second mechanism would involve inhibition of Cx hemichannels, resulting in diminution of the aggregation response through an as yet undefined pathway. It has been proposed, but not yet proven, that Cx hemichannels may act in a similar fashion as Panx1, thus activating P2X_1_ receptors via the release of ATP from intracellular space.

Upon vascular damage, exposure of sub-endothelial collagen and vWF to the blood flow induces the recruitment of platelets in order to promote primary hemostasis at the site of the injury [120]. Adhering platelets then undergo shape change, release messengers including ADP and ATP, and synthetize molecules reinforcing their activation and promoting further platelet recruitment and aggregation [120,121]. Given the crucial role of ADP and ATP release in the activation of purinergic signaling during platelet activation [87], the recent finding that Panx1 is expressed at the surface of human platelets [122,123] paved the way for studies investigating the role of Panx1 channels in platelet aggregation and thrombosis. It was first demonstrated that exposure of platelets to the non-specific Panx1 inhibitors probenecid (Pbn) and carbenoxolone (Cbx) dampened the influx of calcium, subsequent ATP release and aggregation in response to low concentrations of thrombin, collagen or the TxA_2_ analogue U46619 [122]. It was hypothesized that Panx1 plays a role in a granule-independent ATP release amplifying the aggregation response and calcium influx through P2X_1_ receptor [122]. This hypothesis was further strengthened by another independent study demonstrating a physical interaction between Panx1 and P2X_1_ in human platelets [123]. In this study, however, chemical inhibition of Panx1 with the non-specific blockers Pbn and mefloquine (Mfq), or with the specific ^10^Panx1 peptides, decreased ATP release from platelets and their aggregative capacities in response to collagen but not to two other agonists, i.e., ADP and arachidonic acid (Figure 5). These results were confirmed using platelets isolated from *Panx1^−/−^* mice [123]. In addition, a similar inhibition of collagen-induced ATP release and platelet aggregation was observed after pre-incubation of human platelets with the commonly-used food dye Brilliant Blue FCF (Figure 5), which has been shown to inhibit Panx1 channel function in a detailed electrophysiological study [124,125]. Mechanistically, the authors dissected in a series of experiments that collagen-induced activation of GPVI receptors resulted in Panx1 phosphorylation by Src kinase and subsequent opening of the Panx1 channels. Then, ATP would pass through the Panx1 channel towards the extracellular space leading to autocrine/paracrine activation of P2X_1_ receptors and finally platelet aggregation (Figure 5) [123]. The involvement of Panx1 in collagen-induced platelet aggregation was further supported by the finding that a single nucleotide polymorphism (SNP) in the human *Panx1* gene, i.e., *Panx1 400A>C* (rs1138800) encoding for a gain-of-function channel, was associated with increased platelet reactivity to collagen (but not to arachidonic acid and ADP) in healthy subjects [123]. This genotype–phenotype relation was further underscored by a phenotype–genotype relation in a small cohort of patients with an extreme platelet phenotype. Indeed, the gain-of-function *Panx1-400C* polymorph was more frequently found in patients displaying platelet hyper-reactivity than in those showing a hypo-reactive phenotype [123]. Moving from healthy volunteers to a cohort of stable cardiovascular patients resulted unfortunately in loss of the association between the *Panx1* SNP and collagen-induced platelet aggregation [126]. However, it is to be kept in mind that cardiovascular patients compared with healthy controls take different medication including anti-platelet drugs that may blur any fine-tuning effect of the gain-of-function mutation induced by *Panx1-400C*. In addition, it was noted by the authors that the allelic frequency of the *Panx1-400C* allele was significantly different in the cardiovascular patient population as compared to the healthy volunteer cohort (55.5% versus 46.9%, respectively), suggesting that inclusion criteria of the Antiplatelet Drug Resistances and Ischemic Events (ADRIE) study might have favored the recruitment of stable cardiovascular patients with a *Panx1-400C* allele and justifies clinical investigations towards an association between the *Panx1-400A>C* genetic variant and acute ischemic athero-thrombotic disease. Altogether, the selective involvement of Panx1 channels in collagen-induced platelet aggregation makes this channel an attractive new target for platelet inhibition in the context of athero-thrombosis.

### 3.2. Connexins and Platelet Aggregation

Given their essential role in adhesion of epithelial and inflammatory cells [127,128,129,130], the concept that Cxs could participate in mechanisms linked to platelet adhesion, and maybe even aggregation, raised growing interest. The discovery in the 1980s that gap junctions were formed between stromal cells and hematopoietic cells, both in bone marrow tissue and cell culture, suggested that Cxs may be present at the surface of leukocytes and possibly also platelets [131,132,133]. As a confirmation of this possibility, the mRNA of 16 Cxs were found more than 20 years later in megakaryocyctes, among which Cx37, Cx40 and Cx62 showed the highest expression patterns [134]. Some other studies reported the expression of several Cxs in platelets including Cx32, Cx37, Cx40 and Cx43 [134,135,136]. Formation of functional gap junction channels in platelet aggregates was demonstrated by several techniques allowing the tracking of fluorescent dyes (neurobiotin and calcein) that diffuse from one platelet to another [134,135]. Interestingly, blocking or deleting Cx37 prevented the diffusion of this dye. Although Cx32 and Cx43 mRNA have been found in platelets, functional studies on platelet aggregation and thrombus formation are only performed for Cx37 and Cx40. The first study reported in 2011 that Cx37 deletion in mice increased platelet aggregation in response to collagen, thrombin and ADP. Importantly, increased platelet aggregation responses were also observed when a Cx37 mimetic blocking peptide was used to mimic the effects of the knockout in vitro [135]. A subsequent series of experiments let the authors propose that Cx37 forms functional gap junction channels during platelet activation when a stable contact is established, allowing for the diffusion of cAMP from one platelet to another (Figure 5). Through the inhibitory effects of cAMP, further platelet recruitment was suppressed and thrombus growth inhibited. A second group extended the function of Cxs in platelets to hemichannels. They observed that deletion of Cx37 reduced fibrinogen binding as well as granule secretion from platelets even when there was no physical contact between platelets [134], suggesting hemichannel involvement. Moreover, blocking or deleting Cx40 in platelets also decreased α-granule secretion, as indicated by reduced expression of P-selectin [136]. Finally, blocking Cx37 with the specific inhibitor ^37,43^Gap27 in *Cx40^−/−^* murine platelets and inhibiting Cx40 with the blocker ^40^Gap27 in *Cx37^−/−^* platelets further increased aggregation responses, suggesting that the two Cx hemichannels operate independently from each other [136].

It would be interesting to design experiments that can unequivocally distinct the role of Cx37 hemichannels and gap junction channels in platelet aggregation since it appears that mechanisms involving both types of Cx37 channels are able to interfere somewhere in the platelet aggregation cascade. The way that Cx37 hemichannels participate in platelet activation may be closely related to the manner Panx1 channels regulate platelet function. Cx37 hemichannel opening could allow for the release of ATP from activated platelets, which would then act in an autocrine/paracrine fashion on ionotropic P2X_1_ receptors to enhance calcium influx and elevate the level of activation of the platelets (Figure 5). In contrast to Panx1 channels, however, no direct interaction between Cx37 and P2X_1_ has been demonstrated yet. Moreover, the mechanism of Cx37 hemichannel opening in the presence of a relatively elevated extracellular calcium concentration in blood, at which Cx37 hemichannels are normally closed, remains to be established [137].

## 4. “Drug-Ability” of Panx-Cx Targets

Nowadays, the two major anti-platelet drugs prescribed to cardiovascular patients are aspirin and P2Y_12_ inhibitors (clopidogrel, prasugrel, ticagrelor), which respectively target the TxA_2_ and ADP platelet signaling pathways (Figure 5). The use of widely-targeting drugs remains however associated with increased risk of hemorrhage and recurrent ischemic events [138], and the need to find selective-targeting modulators of platelet function is still of importance. Targeting Panx1 channels and possibly Cx channels when the above-described research questions are solved may represent a new potential direction. The following paragraph reviews known pharmacological inhibitors of Panx1 and their potential or proven effects on platelet aggregation (Table 1 and Figure 5).

First, Cbx, a derivative of enoxolone (or glycyrrhetic acid), is an FDA-approved drug used mainly for the treatment of gastric ulcers [139]. It has recently been shown that inhibiting Panx1 with Cbx reduced cancer metastasis in mice [140]. Moreover, Cbx has been shown to inhibit platelet aggregation induced by low doses of collagen (Figure 5) [122]. However, Cbx therapy has been shown to have significant adverse effects such as sodium retention and hypokalaemia [141]. Despite its promising and potent effects on Panx1, Cbx exhibits significant inhibiting effects on Cx channels and may thus be considered non-specific [142].

A second candidate is flufenamic acid, a derivative of fenamate, which has been shown to act as an inhibitor of Panx1 channels [143], although its specificity for Panx over Cxs is still not clear. Flufamenic acid is an inhibitor of the cyclooxygenase pathway and consideration to use this drug to inhibit Panx1 in platelets is no longer valid since a recent study showed that it abolished platelet inhibition by aspirin in humans [144]. Moreover, the use of this drug caused gastrointestinal adverse effects [145].

Mfq, a 4-quinolinemethanol, is a derivative of quinine used as a drug for malaria [146] and a known Panx1 channel blocker. Both quinine and Mfq have been shown to inhibit Panx1 channels, but Mfq is more effective with an IC_50_ of 50 nM [147]. Data on the specificity of both compounds for Panx channels are sparse. The inhibitory effect of quinine on platelet aggregation has been proven some years ago [148] and Mfq was recently shown to inhibit human platelet aggregation and ATP release induced by collagen (Figure 5) [123]. However, the prescription of Mfq is now strongly discouraged as studies have evidenced an association between the use of this drug and severe psychiatric side effects such as anxiety, paranoia, psychosis and anterograde amnesia [149].

Pbn is another candidate compound that is an inhibitor of inorganic acid transporters in many organs, including the kidneys, and is principally used in the treatment of chronic gout and as an additive to increase the levels of antibiotics in the blood [150]. The inhibitory effect of Pbn on platelet aggregation became evident for the first time in the 1990s [151]. In fact, Pbn was reported to inhibit platelet aggregation by affecting cytosolic calcium levels and impeding leukotriene C4 exit [152,153]. However, the discovery that Pbn effects involve a Panx1-dependent mechanism has only been demonstrated recently in two studies showing that pre-treating human platelets with Pbn reduced calcium influx, ATP release as well as aggregation responses (Figure 5) [122,123]. Nevertheless more detailed studies on the potential use of Pbn as an anti-aggregation compound will be needed, as it has been described in a case report that combination treatment with oseltamivir and Pbn induced thrombocytopenia [154].

Finally, the food dye Brilliant Blue FCF, a triarylmethane dye, has been shown in 2013 to act as a specific inhibitor of Panx1 [124]. Due to its strong similarity with the purinergic receptor inhibitor Brilliant Blue G, Brilliant Blue FCF was first thought to act as a chemical blocker of the purinergic receptor P2X_7_, which is known to interact with Panx1 during inflammasome activation [155,156]. It was then demonstrated that no effect of Brilliant Blue FCF was observed, even when high concentrations were used, and that the dye had a specific action on Panx1 with an IC_50_ of 0.27 μM [124]. Moreover, Brilliant Blue FCF does not seem to affect Cx channels, which makes the dye a first choice inhibitor in experimental studies as compared to Cbx, Mfq or Pbn [124]. In a recent study, a relatively high concentration of Brilliant Blue FCF was shown to selectively inhibit collagen-induced aggregation of human washed platelets (Figure 5) [125]. Interestingly, the selectivity for collagen-dependent responses and the non-toxicity of the dye on platelets was underscored by the fact that the dye did not affect the aggregation response to arachidonic acid in these platelets [125]. Additional studies should determine a possible influence of Brilliant Blue FCF on the aggregation induced by other agonists in vitro and on thrombosis in vivo in animal models. Due to its low oral absorption, there are only a few reports on toxicity related to Brilliant Blue FCF, and no evident toxic or carcinogenic effects have been observed in animal models [157,158]. Moreover, low doses of Brilliant Blue FCF are apparently well tolerated in humans as this dye is commonly used in multiple blue food products.

The major issue with Panx1 blockers being their specificity (for Panxs over Cxs), mimetic peptides have been developed for research purposes. ^10^Panx1 peptides have been shown to selectively reduce collagen-induced aggregation of human washed platelets by about 40%, while a scrambled peptide did not display any effects. Similarly, no effects were seen on the aggregation response induced by other agonists (Figure 5) [123]. ^10^Panx1 is a mimetic peptide of the first EL domain of Panx1 that impedes the passage of small molecules and ATP through the channel [159]. Although controversial and needing further confirmation in other expression systems, we should keep in mind that ^10^Panx1 was shown to inhibit the lens-specific Cx46 hemichannel currents in oocytes to about the same extent as Panx1 currents [160]. There is, however, a more promising development involving peptide-based inhibition of Panx1 channels that may also be suitable for platelets [161,162,163]. The peptide contains a TAT sequence allowing its entry into the cell. Once in the cytoplasm, the peptide recognizes the binding site (Y308) for Src proteins on Panx1 and avoids the phosphorylation of the protein, thus inhibiting channel opening. The efficacy of this strategy has been demonstrated in a study where inhibition of NMDA-dependent Panx1 phosphorylation by Src was blocked by the TAT-tagged peptide (TAT-Panx1_308_) and conferred a significant neuroprotection before or two hours after ischemic stroke in adult rats [161]. This peptide-based strategy requires urgent testing in the context of platelet aggregation as it may represent a new manner to selectively modulate collagen-induced platelet aggregation that also involves Src-dependent phosphorylation of Panx1 channels [123]. Indeed, research towards compounds selectively inhibiting Panx1 channels is only in its infancy but holds fair promise for the development of selective inhibitors of collagen-induced platelet aggregation.

## 5. Conclusions

The role of Cxs and Panx1 in platelet aggregation is an exciting field of actual research, which holds promise for targeted drug development in order to prevent thrombotic diseases. Future investigations should provide further detailed insight into the molecular mechanisms linking Cx hemichannels or gap junctions to platelet reactivity. Our mechanistic insight into the role of Panx1 channels in platelet reactivity is rather advanced and already allows for a careful search towards new pharmacological strategies aiming at reducing ischemic events in cardiovascular patients without increasing the risk of bleeding.

## Figures and Tables

**Table 1:** Effects of Panx1 inhibitors on platelet aggregation.

Compound’s Name	Clinical Applications	Effects on Platelet Aggregation	Possible Side Effects
Carbenoxolone	Gastric ulcer [139]	Reduces collagen-induced aggregation [122]	Sodium retention, hypokalaemia [141]
Flufenamic acid	NA ^1^	Reduces aggregation via COX inhibition [144]	Aspirin antagonism [144], GI ^2^ disturbances [145]
Mefloquine	Malaria [146]	Reduces collagen-induced aggregation [123]	Psychiatric diseases [149]
Probenecid	Gout; Adjuvant for antibiotics [150]	Inhibits aggregation [122,123,152,153]	Thrombocytopenia [154]
Brilliant blue FCF	NA	Reduces collagen-induced aggregation [125]	NA
^10^Panx1 peptide	NA	Reduces collagen-induced aggregation [123]	NA
TAT-Panx1^308^	NA	NA	NA

^1^ NA (non applicable); ^2^ GI (gastrointestinal).

## Abbreviations

AMPA	α-Amino-3-hydroxyl-5-methyl-4-isoxazole-propionate
NMDA	*N*-methyl-d-aspartate
GP	Glycoprotein
vWF	Von Willebrand
PAR	Protease-activated receptor
cAMP	Cyclic adenosine monophosphate
FcR	Fc receptor
ITAM	Immunoreceptor tyrosine-based activation motif
Syk	Spleen tyrosine kinase
PLCγ2	Phospholipase Cγ2
PI3	Phosphoinositide-3
IP3	Inositol 1,4,5-trisphosphate
DAG	Diacylglycerol
PKC	Protein kinase C
TxA_2_	Thromboxane A_2_
PIP3	Phosphatidylinositol 3,4,5-trisphopsphate
CRP	Collagen-related peptide
MIDAS	Metal ion-dependent adhesion site
TF	Tissue factor
RIAM	Rap1-GTP-interacting adaptor molecule
ADP	Adenosine diphosphate
CD40L	CD40 ligand
SFK	Src family kinase
PI3-K	Phosphoinositide 3-Kinase
ATP	Adenosine trisphosphate
SLAM	Signaling lymphocyte activation molecule
Panx	Pannexin
Cx	Connexin
TM	Transmembrane
NT	Amino-terminal
CT	Carboxy-terminal
EL	Extracellular loop
IL	Intracellular loop
ER	Endoplasmic reticulum
Pbn	Probenecid
Cbx	Carbenoxolone
Mfq	Mefloquine
SNP	Single nucleotide polymorphism
ADRIE	Antiplatelet Drug Resistances and Ischemic Events
